# A new frontier of image guidance: Organs at risk avoidance with MRI‐guided respiratory‐gated intensity modulated radiotherapy: Technical note and report of a case

**DOI:** 10.1002/acm2.12575

**Published:** 2019-05-04

**Authors:** Mariangela Massaccesi, Davide Cusumano, Luca Boldrini, Nicola Dinapoli, Bruno Fionda, Stefania Teodoli, Luigi Azario, Gian Carlo Mattiucci, Mario Balducci, Francesco Cellini, Vincenzo Valentini

**Affiliations:** ^1^ Dipartimento di Diagnostica per Immagini UOC di Radioterapia Oncologica Radioterapia Oncologica ed Ematologia Fondazione Policlinico Universitario “A. Gemelli” IRCCS Rome Italy; ^2^ Dipartimento di Diagnostica per Immagini UOC di Fisica Sanitaria Radioterapia Oncologica ed Ematologia Fondazione Policlinico Universitario “A. Gemelli” IRCCS Rome Italy; ^3^ Istituto di Radiologia Università Cattolica del Sacro Cuore Rome Italy; ^4^ Istituto di Fisica Università Cattolica del Sacro Cuore Rome Italy

**Keywords:** adaptive radiotherapy, hybrid radiotherapy, MRI guided radiotherapy, respiratory gating

## Abstract

The case of a 50‐year‐old man affected by a rhabdomiosarcoma metastatic lesion in the left flank Is reported. The patient was addressed to 50.4 Gy radiotherapy with concomitant chemotherapy in order to locally control the lesion. A Tri‐60‐Co magnetic resonance hybrid radiotherapy unit was used for treatment delivery and a respiratory gating protocol was applied for the different breathing phases (Free Breathing, Deep Inspiration Breath Hold and Final Expiration Breath Hold). Three intensity modulated radiation therapy (IMRT) plans were calculated and Final Expiration Breath Hold plan was finally selected due to the absence of PTV coverage differences and better organs at risk sparing (i.e. kidneys). This case report suggests that organs at risk avoidance with MRI‐guided respiratory‐gated Radiotherapy is feasible and particularly advantageous whenever sparing the organs at risk is of utmost dosimetric or clinical importance.

## 1 INTRODUCTION

Several sources of geometric uncertainties exist in the radiotherapy process. Tumor motion represents a major source of uncertainty. Particularly, breathing‐induced tumor motion up to 2 cm is common in lung and upper abdomen and even larger excursions can occur.^1^, ^2^, ^3^ To ensure an adequate coverage of the tumor with the intended dose, a margin for intra/inter‐fraction patient changes (internal margin) and for setup uncertainties (setup margin) is added around the clinical target volume to obtain the planning target volume (PTV). Strategies to account for this motion while minimizing PTV include breath hold or free‐breathing (FB) respiratory tracking and gating techniques. In particular, breath hold technique can be guided by supporting the patient with dedicated systems, such as the active breathing co‐ordinator system (Elekta, Stockholm, Sweden). All these methods require a technological link between the detection of tumor position during the breathing cycle and treatment delivery. For this purpose, invasively implanted fiducial markers are available but they are associated with additional risks, costs, and require specific expertise. Fiducial‐less solutions based on surrogates of tumor motion, such as the body surface or the diaphragm, have been proposed; variations in the correlation between tumor movements and surrogate signals can lead, however, to uncertainties resulting in a poor treatment outcome. Real‐time cine planar magnetic resonance imaging (MRI) offers the unique possibility to directly detect and gate the treatment to a particular condition of the respiratory cycle eliminating the need for surrogate‐based systems and imaging dose to patient. MRI‐guided gated treatments can be performed during either breath hold or FB, depending on patient compliance and organs at risk (OARs) irradiation which may vary during the different conditions of the breathing cycle.^4^ Even though a particular tumor may not show significant respiratory induced motion, remarkable changes may occur in the position of the surrounding OARs during the breathing cycle: this particular situation generally occurs when the target lesions are fixed (i.e., infiltrating tumors such as sarcomas) or when spinal or bone metastases (more specifically of the axial skeleton) are treated. In such cases, gating the radiation treatment to the position of an OAR might offer a possibility for a further personalization of the treatment delivery.

The MRIdian^®^ system (ViewRay Inc., Cleveland, OHo, USA) is a hybrid machine that consists of two main components: a 0.35 Tesla MRI scanner and a radiation delivery system, composed either by a set of three Cobalt‐60 (tri‐Co‐60) sources or a 6 MV linear accelerator, in its recently released MRI‐Linac version.^5^

The MRIdian^®^ system offers an integrated solution for online adaptive radiotherapy, allowing to modify the dose distribution taking into account the morphological changes occurring among different therapy days.^6^, ^7^ In the next future, the online replanning could also be carried out on the basis possibly biological variations highlighted through advanced imaging analysis and diffusion weighted MRI sequences.^8^, ^9^

A case where MRI‐guidance has been successfully used to reduce normal tissue irradiation by performing the gating on an OAR is described in this report. To the best of our knowledge, this is the first study demonstrating the technical feasibility of this approach.

## 2 CASE DESCRIPTION

**Figure 1:**
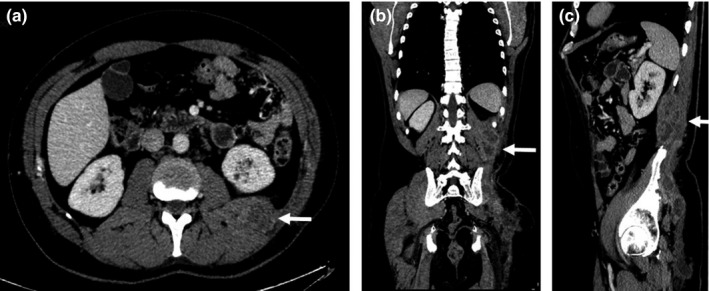
Computed tomography images showing an inhomogenous mass in the left flank of the patient extending through the retroperitoneal fat toward the peri‐renal space (white arrows) in axial (a), coronal (b), and sagittal (c) views.

A 50‐year‐old man with a large metastatic lesion of a primary rhabdomyosarcoma in the left flank region was treated. He had first been diagnosed with a rhabdomyosarcoma in February 2016 with a primary 12 localization in the left gluteus muscle. The patient received surgery, postoperative high dose rate brachytherapy (25 Gy in 5 fractions) and adjuvant chemotherapy. In September 2016 a restaging Positron emission tomography–computed tomography (PET‐CT) imaging revealed two metastatic lesions, both in the thorax. The first lesion, located in the lung, was surgically removed and histologically confirmed. The second one, located in the para‐aortic space, was treated with stereotactic radiotherapy (total delivered dose 40 Gy in 5 fractions with linear accelerator through volumetric modulated arc technique, VMAT). In May 2017 the patient developed a local recurrence (left gluteus) and underwent re‐resection. In August 2017 a contrast enhanced total body CT scan showed a large tumor mass within the contest of the paravertebral muscles in the left flank measuring 7 × 6 × 10 cm (Fig. 1). A new course of radiotherapy up to a total dose of 50.4 Gy in 28 fractions was prescribed with concurrent chemotherapy to limit the tumor mass growth.

## 3 TREATMENT DESCRIPTION

### 3.1 Setup and treatment planning

The patient was immobilized using the FluxBoardTM device (MacroMedics^®^, Amsterdam, The Netherlands), which allowed indexing of patient positioning and facilitated MR coils placement. A helical CT scanner (HiSpeed 26 DX/i 26 Spiral, General Eletrics, Fairfield, CT, USA) was used for simulation CT imaging acquisition (slice thickness was 2.5 mm). The patient was scanned in supine position. No bowel preparation neither intravenous contrast was applied. Three CT scans were acquired to judge which breathing condition ensures the best anatomy separation between target and OARs: a four‐dimensional (4D) CT scan during FB, and two‐three–dimensional CT scans during either deep inspiration breath hold (DIBH) or final expiration breath hold (FEBH). Three MRI scans were then obtained in the same breathing conditions (FB, DIBH, and FEBH) on the MRIdian system, using a true fast imaging with steady state precession (TRUFI) without administering any contrast medium. The slice thickness was 1.5 mm and the acquisition time was 17 s. The FB acquisition was averaged across the acquisition time and a motion artifact on the anterior part of the abdomen was visible. During the MRI simulation, a sagittal cine planar MRI scan was acquired during FB over a period of 120 s to estimate the breathing induced motion of the tumor, that was found to be negligible (<2 mm maximum amplitude).

The gross tumor volume (GTV) and OARs (right and left kidney, cauda, bowel as both whole intestinal cavity and bowel loops) were delineated on the CT images registered with the corresponding MRI images in each breathing condition on the MRIdian treatment planning system (v. 61 4.5.1.239).

The average intensity projection has been used for FB planning, while the aforementioned DIBH and FEBH CT scans for the respective respiratory condition. The GTV was expanded by 5 mm in all directions to create a PTV to account for delineation error, internal motion, and setup error.

Considering the negligible movement observed for the primary lesion during the simulation condition and taking into account the foreseen maintenance chemotherapy that the patient would undergo after the radiochemotherapy treatment, we decided to preserve as much as possible the surrounding organs at risk and in particular the omolateral kidney, in order to reduce the risk of potential renal complications related to the expected further treatments.

Volumetric values for left kidney, GTV, and PTV for FB, DIBH, and FEBH plans are reported in Table 1.

**Table 1:** Volumetric values (cm^3^) for left kidney, gross tumor volume (GTV), and planning target volume (PTV) for free breathing (FB), deep inspiration breath hold (DIBH), and final expiration breath hold (FEBH) plans

	FB	DIBH	FEBH
Left kidney	240.9	220.1	269.3
GTV	630.4	635.0	630.9
PTV	855.6	852.4	867.1

Three intensity modulated radiation therapy (IMRT) plans were calculated (FB, DIBH, and FEBH) and compared one to each other. Dose calculation was performed by using a Monte Carlo–based algorithm, setting a grid resolution of mm^3^ and taking into account the presence of the magnetic field. The dose distribution was optimized in order to obtain the highest PTV coverage while maintaining the dose to the homolateral kidney and the small bowel as low as reasonably achievable. All treatment plans were normalized setting 50.4 Gy as the mean target dose.

Three intensity modulated radiation therapy (IMRT) optimization parameters have been intentionally set in the same way for all the plans (IMRT efficiency = 2.5; Levels = 7) in order to guarantee a fair comparability among the plans.

**Figure 2:**
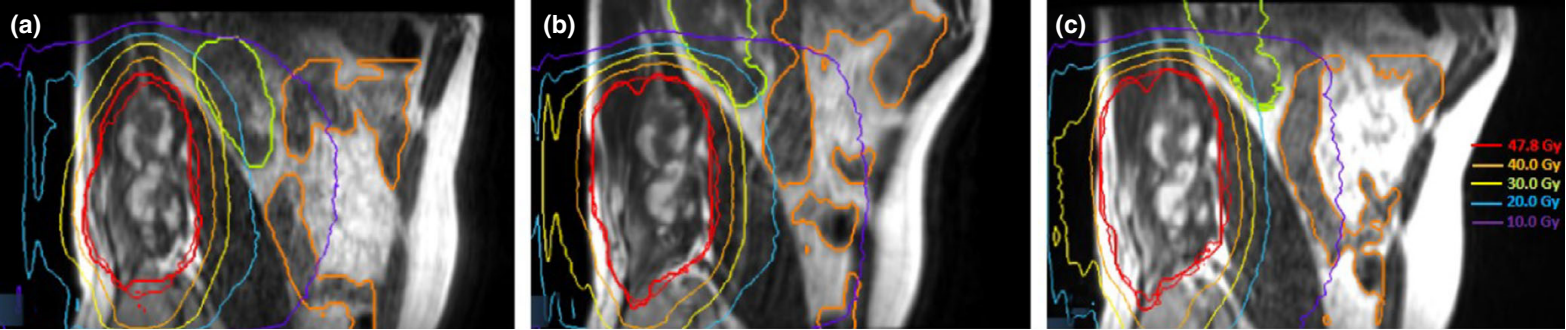
Sagittal views of planning magnetic resonance imaging (MRI) MRIdian true fast imaging with steady state precession sequences with dose distribution in deep inspiration breath hold (a), free breathing (b), and final expiration breath hold (c) conditions.

Dose distributions (Fig. 2) and dose volume histograms (DVHs) were computed and compared to choose the best plan. Estimated beam on times were 112, 84, and 248 s for FB, DIBH, and FEBH plan, respectively.

Deformable summation of dose to prior radiation plan was not performed due to uncertainties of deformable registration between the varying breathing conditions.

In the absence of clear PTV coverage differences, FEBH plan was finally selected due to better OARs sparing, prioritizing the avoidance of the kidneys (Dmean) and more specifically of the omolateral (left) one.

Table 2 reports all the dosimetric parameters taken into account for respiratory condition selection.

**Table 2:** Dose volumetric parameters for organs at risks (OARs; lower values in bold font) and planning target volume (PTV)

	FB	DIBH	FEBH
Left kidney			
Dmax (Gy)	49.8	49.0	**48.3**
Dmean (Gy)	16.05	22.99	**13.39**
V5 (cc)	**173.9**	220.1	182.9
V10 (cc)	**140.9**	219.7	149.8
V20 (cc)	85.6	127.1	**60.3**
V30 (cc)	35.5	39.8	**9.2**
V40 (cc)	11.8	8.6	**5.4**
V50 (cc)	0	0	0
Kidneys			
Dmean (Gy)	11.34	16.04	**9.71**
Intestinal cavity			
Dmax (Gy)	49.7	**32.5**	38.2
V5 (cc)	1867.4	2682.9	**1605.2**
V10 (cc)	749.5	1019.3	**230.4**
V20 (cc)	80.7	29.9	**4.7**
V30 (cc)	18.3	0.16	**0.2**
V40 (cc)	5	0	0
V50 (cc)	0	0	0
Bowel loops			
Dmax (Gy)	36.6	27.5	**24.4**
V5 (cc)	1011.5	1461.1	**687.8**
V10 (cc)	392.4	645	**131.3**
V20 (cc)	39.4	9.4	**1.2**
V30 (cc)	1.5	0	0
V40 (cc)	0	0	0
V50 (cc)	0	0	0
PTV			
V95 (%)	95.47	95.15	95.59
V105 (%)	3.95	4.02	3.87

FB, free breathing; DIBH, deep inspiration breath hold; FEBH: final expiration breath hold.

### 3.2 Treatment delivery

A daily setup 0.35 T MRI was acquired during FEBH at 1.5 mm isotropic spatial resolution and 17 s acquisition time. The MR images were registered by the attending physician with the treatment planning CT using a manual imaging registration method focusing on the GTV. After image fusion was satisfactorily accomplished, the couch was repositioned and a verification cine MRI in FEBH was performed over a period of 17 s before treatment delivery to check the reproducibility of the breathing condition. To this purpose the left kidney was selected as target structure for the gating giving an isotropic 4 mm boundary and gating the treatment to no more than 5% of the kidney out of boundaries. For MR‐based real‐time tracking of the sagittal plane, boundaries of 3–5 mm beyond the tracking structure have shown to be feasible with % ROI out of 2‐10% for gating criteria.^10^, ^11^ The lesion was placed at the center of the cine field of view to ensure the highest spatial integrity of the image.^12^

The chosen boundary values (4 mm) have been set starting from the measurements performed on a FEBH cine‐MRI which showed that the left kidney lied in a 4 mm boundary area for more than 95% of the acquisition time.

The feasibility of the delivery settings (in terms of gating boundary and kidney out of boundary percentage) was tested during a dedicated preview analysis performed prior to each fraction: if the simulated beam on time resulted to be more than 70% of the monitored time with the chosen parameters, the treatment proceeded; otherwise, the setup verification process had to be repeated. No online adaptations were performed in the treatment of this patient.

**Figure 3:**
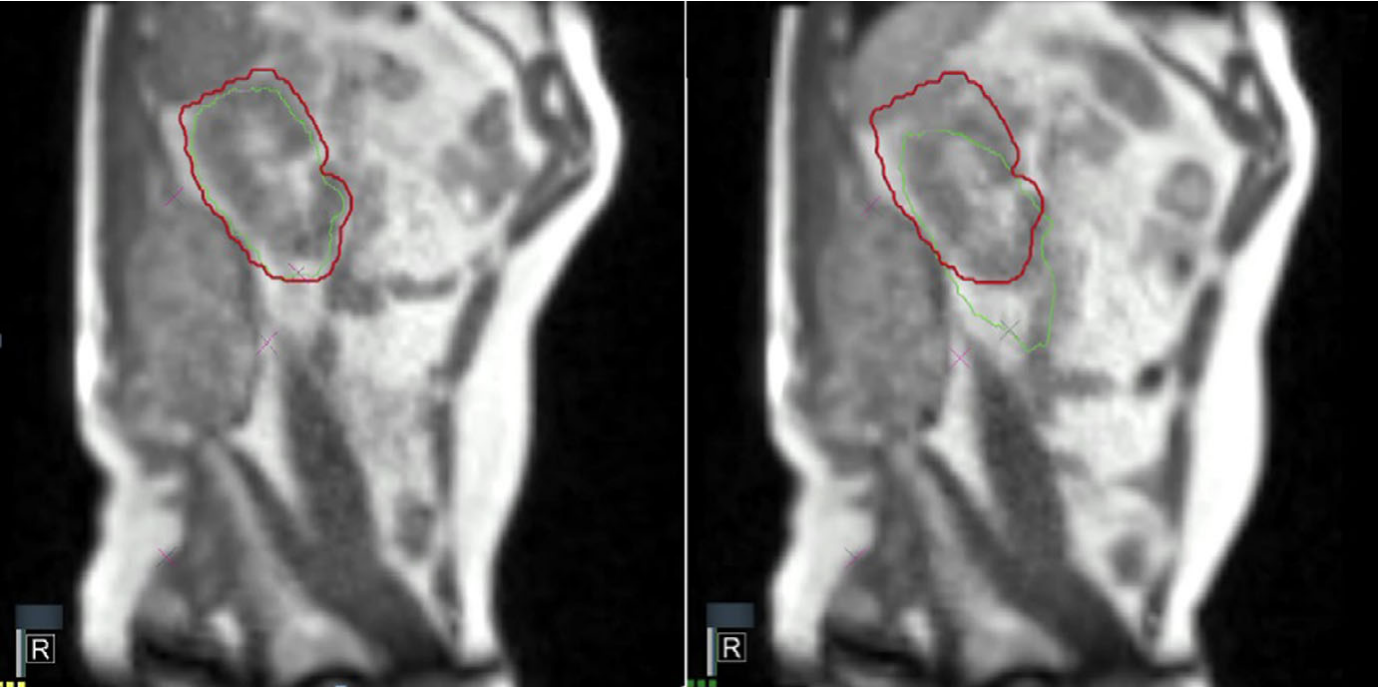
Two‐dimensional magnetic resonance treatment cine views: the kidney (red) and the boundary area (green) are contoured and shown in beam on (left) and beam off (right) conditions.

Figure 3 shows two frames of the two‐dimensional (2‐D) MR cine during the treatment delivery: in the left part, the kidney is in the correct treatment position for beam on, in the right part the kidney is out of the treatment position and the beam is off.

During treatment delivery the patient had the possibility to look at a monitor displaying in real time the actual position of his left kidney relatively to its ideal position and adjust the depth of his breath to maintain the right position as long as possible.

Lamb et al. have recently discussed the latency of the gating process implemented by MRIdian, whose maximum beam‐off latency is equal to 0.5 s, as specified by the manufacturer.^13^

To limit the dosimetric effect of the gating latency, the number of transitions between gated and ungated states was limited, giving a sufficient time to the patient to recover from each breath hold inspiration.

The patient completed all fractions without unintended interruptions. The median duration of each fraction was 25 min and 32 s (range: 21 min 23 s–40 min 52 s) including imaging and treatment delivery.

The tumor size remained stable for 6 months, after that the mass started to re‐growth. No radiation‐induced toxicities were observed during‐treatment and during the follow‐up period.

The sagittal plane chosen for cine‐MRI acquisition was manually defined, selecting the slice in which the distance between the two centers of mass (COM) of the PTV and the left kidney was minimal. The adequate target coverage for the single fractions was assured using the dose prediction tool of the MRIdian treatment planning system.

## 4 CONCLUSION

Organs at risk avoidance with MRI‐guided respiratory‐gated tri‐60‐Cobalt Intensity Modulated Radiotherapy is feasible and relatively easy to implement. The described procedure appeared to successfully deal with organ motion, as the MRI has the advantage of displaying soft tissues with better contrast and higher anatomical detail as compared to the usual on‐board kV or cone beam CT (CBCT) imaging systems that provide only rough visualization of the therapy volumes or their surrogates (such as the diaphragm for respiratory movements).

Besides these technical advantages, the principal rationale of using this approach was the observation of a sound overall dosimetric benefit using a specific breathing condition for planning purposes.

The use of a gating approach on a movable OAR allowed indeed to maximize organ sparing, ensuring a clinical advantage that appeared not to be reachable in the other considered breathing conditions (FB and DIBH), while maintaining in the same time an optimal target coverage. Further advantages of this technique are represented by the high time resolution (unlike the 4D CBCT, which typically requires at least a single 1 min gantry rotation to acquire) and the possibility to not expose patients to unnecessary radiation.

The approach we used also improved the treatment's dosimetric outcome as we demonstrated that directly tracking the target volume is not always the most suitable planning solution and that the described irradiation technique could therefore be particularly advantageous whenever sparing the organs at risk is of utmost importance, such as in stereotactic treatments and re‐irradiation, however, in case of retreatment the calculation of dose accumulation may be hampered by the difference of breathing conditions.

## CONFLICT OF INTEREST

Dr. D. Cusumano, Dr. L. Boldrini, Dr. N. Dinapoli, and Dr. F. Cellini have received speaker honoraria and travel reimbursements from ViewRay Inc. Prof. V. Valentini has a research agreement with ViewRay Inc.
